# Functional analysis of rare anti-Müllerian hormone protein-altering variants identified in women with PCOS

**DOI:** 10.1093/molehr/gaad011

**Published:** 2023-04-02

**Authors:** L Meng, A McLuskey, A Dunaif, J A Visser

**Affiliations:** Department of Internal Medicine, Erasmus MC, University Medical Center Rotterdam, Rotterdam, The Netherlands; Department of Internal Medicine, Erasmus MC, University Medical Center Rotterdam, Rotterdam, The Netherlands; Division of Endocrinology, Diabetes and Bone Disease, Department of Medicine, Icahn School of Medicine at Mount Sinai, New York, NY, USA; Department of Internal Medicine, Erasmus MC, University Medical Center Rotterdam, Rotterdam, The Netherlands

**Keywords:** AMH, anti-Müllerian hormone, PCOS, variants, processing, signaling

## Abstract

Recently, rare heterozygous *AMH* protein-altering variants were identified in women with polycystic ovary syndrome (PCOS), causing reduced anti-Müllerian hormone (AMH) signaling. However, the exact functional mechanism remains unknown. Here, we analyzed the processing, secretion, and signaling of these AMH variants. Functional analysis of six PCOS-specific AMH variants (V^12^G, P^151^S, P^270^S, P^352^S, P^362^S, H^506^Q) and one control-specific variant (A^519^V) was performed in the mouse granulosa cell-line KK-1. Human (h) AMH-^151^S and hAMH-^506^Q have ∼90% decreased AMH signaling compared to wild-type (wt) AMH signaling. Coexpression of hAMH-^151^S or hAMH-^506^Q with wt-hAMH dose-dependently inhibited wt-hAMH signaling. Western blotting revealed that hAMH-^151^S and hAMH-^506^Q proteins were detected in the cell lysate but not in the supernatant. Confocal microscopy showed that HEK293 cells expressing hAMH-^151^S and hAMH-^506^Q had higher cellular AMH protein levels with endoplasmic reticulum (ER) retention compared to cells expressing wt-hAMH. Using two AMH ELISA kits, hAMH-^151^S was detected in the cell lysate, while only very low levels were detected in the supernatant. Both hAMH-^362^S and hAMH-^519^V were detectable using the automated AMH ELISA but showed severely reduced immunoactivity in the manual ELISA. Surprisingly, hAMH-^506^Q was undetectable in both the cell lysate and supernatant using either ELISA. However, in PCOS cases, heterozygous carriers of the P^151^S and H^506^Q variants still had detectable AMH in both assays. Thus, P^151^S and H^506^Q disrupt normal processing and secretion of AMH, causing ER retention. Additionally, AMH variants can impair the AMH immunoactivity. An AMH variant may be considered when serum AMH levels are relatively low in PCOS cases.

## Introduction

Polycystic ovary syndrome (PCOS) is a common endocrine disorder and a leading cause of infertility among women of reproductive age. PCOS has a prevalence of 10–15% worldwide, which may even increase to over 25% in severely obese women (Kataoka *et al.*, 2019). According to the Rotterdam criteria, the diagnosis of PCOS is based on the presence of at least two of the following three characteristics: hyperandrogenism; oligo- or amenorrhea; and polycystic ovary morphology (Fauser *et al.*, 2004). In addition to being a reproductive disorder, PCOS also presents as a lifelong metabolic disorder, as women are often obese and have an increased risk for type 2 diabetes (Rodgers *et al.*, 2019). PCOS is highly heritable and ∼20 susceptibility loci have been reproducibly mapped to common genetic variants that implicate neuroendocrine, reproductive, and metabolic pathways in PCOS pathogenesis (Dapas and Dunaif, 2022). *In utero* exposure to testosterone can produce phenotypes of PCOS in a variety of animal models (Stener-Victorin *et al.*, 2020). Recent studies suggest that anti-Müllerian hormone (AMH) signaling also plays a role in the pathophysiology of PCOS (Moolhuijsen and Visser, 2020).

AMH is a member of the transforming growth factor β (TGFβ) family and is mainly expressed by the granulosa cells (GCs) of preantral and antral follicles in the ovary (Weenen *et al.*, 2004). Serum AMH concentrations correlate with the number of growing follicles, and in women with PCOS, this results in a 2- to 4-fold increase in serum AMH levels compared to normo-ovulatory women (Fallat *et al.*, 1997; Laven *et al.*, 2004). Furthermore, an increased AMH production per follicle may further contribute to elevated serum AMH levels in PCOS (Bhide *et al.*, 2015). In the ovary, AMH acts as a gatekeeper of follicle growth as it inhibits follicular atresia, FSH sensitivity of small antral follicles, and FSH-induced aromatase activity (Weenen *et al.*, 2004; Visser and Themmen, 2014). Thus, it has been suggested that in PCOS, the increased AMH levels may contribute to the follicular arrest and increased androgen production (Moolhuijsen and Visser, 2020). Recent studies in mice have shown that AMH also has extragonadal effects as it increases GnRH-mediated LH pulsatility and secretion by direct actions on the hypothalamus and it inhibits *Cyp19a1* expression, encoding aromatase, in the placenta (Tata *et al.*, 2018). Interestingly, *in utero* AMH exposure of mice at the end of the gestation period resulted in a PCOS-like phenotype in adult female offspring (Tata *et al.*, 2018). The extragonadal effects of AMH lead to maternal hyperandrogenism, explaining the observed phenotype in this mouse model, as recently shown by Ho *et al.* (2021). Based on these studies, it is suggested that elevated AMH not only exaggerates the PCOS phenotype but also may cause PCOS.

In contrast, two recent studies suggest that reduced AMH signaling may contribute to the PCOS etiology. Gorsic *et al.* (2019) and Gorsic *et al.* (2017) identified functional PCOS-specific heterozygous rare coding, as well as noncoding, variants in the *AMH* and AMH-specific type 2 receptor (*AMHR2*) genes in ∼7% of the women of their European ancestry PCOS cohort. *In vitro* analysis showed that the *AMH* protein-altering variants resulted in reduced AMH signaling in a dominant negative manner. However, the exact functional mechanisms by which these variants exert this dominant negative effect remain unknown.

AMH is produced as an inactive homodimeric precursor containing a N-terminal pro-region (110 kD) and a smaller C-terminal mature domain (25 kD) (Pierre *et al.*, 2016). Upon or after secretion, the homodimeric AMH precursor (proAMH) undergoes cleavage at a monobasic cleavage site to generate the bioactive protein (Nachtigal and Ingraham, 1996; di Clemente *et al.*, 2010). Upon cleavage, the pro-region (AMH_N_) and mature domain (AMH_C_) remain associated as a noncovalent form (AMH_N,C_) until binding to the receptor complex (di Clemente *et al.*, 2010; Pierre *et al.*, 2016; Hart *et al.*, 2021). The mature domain drives the signaling and biological activity of AMH (Visser and Themmen, 2014). Therefore, proper AMH processing, secretion as well as cleavage are crucial steps to allow normal AMH signaling. Like other members of the TGFβ family, AMH signaling is mediated through a heterodimeric serine/threonine kinase receptor complex, containing the AMH-specific type II receptor (AMHR2) and type I receptors, mainly activin receptor-like kinase (ALK)2 and 3 that are shared with bone morphogenetic protein (BMP) ligands. Activation of this receptor complex by AMH induces the intracellular BMP-like SMAD pathway, resulting in the phosphorylation of the downstream SMAD1, 5, or 8 proteins (reviewed in Visser and Themmen (2014)).

In this study, we have therefore analyzed the processing, secretion, and signaling of six previously reported PCOS-specific rare AMH variants (V^12^G, P^151^S, P^270^S, P^352^S, P^362^S, H^506^Q), selected based on a range of decreased signaling activity. We also included an AMH variant identified in control women, without PCOS, (A^519^V) as this variant is close to an antibody-epitope recognition site.

## Materials and methods

### Generation of AMH expression constructs

Quick-change site-directed mutagenesis was used according to the protocol of Stratagene (Agilent Technologies Netherlands BV, Amstelveen, The Netherlands) to introduce the PCOS-specific variants V^12^G, P^151^S, P^270^S, P^352^S, P^362^S, H^506^Q or the control variant A^519^V into the pcDNA3.1-*human AMH* (*hAMH*) cDNA expression plasmid containing either a wild type (*hAMH-RAQR*) or optimized cleavage site (*hAMH-RARR*), which allows for efficient cleavage of AMH (Weenen *et al.*, 2004). In addition, we generated an hAMH expression plasmid containing a cleavage-resistant site (*hAMH-RAGA*), shown to be normally secreted (Nachtigal and Ingraham, 1996). The DNA of all expression constructs was sequenced to verify the presence of the desired variant.

### Cell transfections

The mouse GC line KK-1 has previously been shown to be a suitable model to study AMH signaling of AMH variants, in part owing to low basal BRE-luciferase reporter activity (Kevenaar *et al.*, 2008; Hoyos *et al.*, 2020). Therefore, KK-1 cells, stably transfected with an AMHR2 expression plasmid, were used to analyze AMH-induced luciferase activity, as described previously with slight modifications (Kevenaar *et al.*, 2008; Hoyos *et al.*, 2020). Cells were cultured in DMEM/F12 (Life Technologies, Inc., Invitrogen, Breda, The Netherlands) with 10% v/v fetal calf serum (FCS, Life Technologies). Fugene HD transfection reagent (Promega, Benelux, Leiden, The Netherlands) was used as transfection reagent.

#### Experiment 1

KK-1 cells were seeded at 70% confluency in a T25 culture flask and transfected with the BRE-Luc reporter plasmid (2 µg) (Korchynskyi and ten Dijke, 2002), the pRL-SV40 plasmid (1 µg) (internal control for transfection efficiency), together with the hAMH variants expression plasmids (1 µg). Twenty-four hours after transfection, cells were seeded into 24-well plates. Three independent experiments were performed in triplicate.

#### Experiment 2

KK-1 cells were plated in a 48-well plate (4 × 10^4^ cells/well) (Greiner Bio-One, Rotterdam, The Netherlands). Cells were transfected with the BRE-Luc reporter plasmid (100 ng), the pRL-SV40 plasmid (25 ng), wild-type (wt)-hAMH expression construct (50 ng), and increasing amount of the hAMH variant expression constructs. The empty pcDNA3.1 plasmid was used to yield a similar total amount of DNA for transfection.

For both experiments 1 and 2, ∼24 h following transfection, cells were cultured in medium containing 0.2% FCS for 24 h, followed by luciferase activity measurement using the Dual-Glo luciferase assay (Promega, Benelux, Leiden, The Netherlands). Independent experiments were performed three to six times in triplicate.

#### Experiment 3

KK-1 cells were plated in a 48-well plate (4 × 10^4^ cells/well) (Greiner Bio-One). Cells were transfected with the BRE-Luc reporter plasmid (100 ng) and the pRL-SV40 plasmid (25 ng). After ∼24 h following transfection, cells were stimulated overnight with increasing concentrations of exogenous wt-hAMH in 0.2% FCS medium.

#### Experiment 4

KK-1 cells were plated in a 48-well plate (4 × 10^4^ cells/well) (Greiner Bio-One). Cells were transfected with the BRE-Luc reporter plasmid (100 ng), the pRL-SV40 plasmid (25 ng), and hAMH-^151^S or hAMH-^506^Q expression plasmids (300 ng). Following transfection, cells were incubated overnight with 5 ng/ml exogenous wt-hAMH in 0.2% FCS medium.

For both experiments 3 and 4, 24 h following the exogenous wt-hAMH incubation, luciferase activity was measured using the Dual-Glo luciferase assay (Promega). Independent experiments were performed three to four times in triplicate.

### Recombinant AMH production

Previously, we have produced recombinant hAMH using human embryonic kidney (HEK) epithelial 293 cells, as these cells have a high efficiency of producing recombinant proteins and contain proprotein convertases, such as Furin, necessary for the cleavage of AMH (Kevenaar *et al.*, 2008; Hoyos *et al.*, 2020). Therefore, we have used HEK293 cells to assess the processing and secretion of these rare AMH variants. HEK293 cells were stably transfected with hAMH-^151^S, hAMH-^352^S, hAMH-^362^S, hAMH-^506^Q, hAMH-^519^V, or wt-hAMH expression constructs. Cell lysates and supernatants were collected from the stably transfected cells under serum-free culture conditions, as described previously (Kevenaar *et al.*, 2008).

### Western blot analysis

Total proteins from conditioned medium or isolated from cell lysates were separated using polyacrylamide gel electrophoresis (12% acrylamide) under reducing conditions, as previously described (Kevenaar *et al.*, 2008; Hoyos *et al.*, 2020) In brief, blots were incubated with the mouse monoclonal antibodies 5/6A (recognizing the C-terminal mature region) at a 1:1000 dilution (Serotec, Bio-Rad Laboratories BV, Veenendaal, The Netherlands), followed by the Alexa Fluor-800 goat anti-mouse antibody (Molecular Probes, Invitrogen, Breda, The Netherlands) at a 1:7500 dilution. The Licor-Odyssey imaging system was used for protein visualization and blots were analyzed using the Odyssey software version 5.2 (LI-COR Biosciences, Westburg, Leusden, The Netherlands).

### Immunofluorescence staining

HEK293 cells, stably transfected with hAMH variant expression constructs, were seeded in an eight-well chamber slide (0.5 × 10^5^ cells/well) (Nunc™ Lab-Tek™ II Chamber Slide™ System, Thermo Scientific, Waltham, MA, USA). After 24 h, cells were washed, fixed and permeabilized with cold methanol for 10 min at −20°C. After rinsing with PBS, cells were blocked with 5% goat serum (Vector Laboratories, Burlingame, CA, USA) in PBS for 1 h at room temperature. Cells were then incubated overnight at 4°C in a humidified chamber with the respective primary antibodies (AMH 5/6A, 1:200; Calreticulin, 1:500, cat. no. ab92516, Abcam (Abcam Netherlands BV, Amsterdam, The Netherlands); GM130, 1:250, cat.no. ab52649, Abcam) diluted in PBS-BSAc (Aurion, Wageningen, The Netherlands), followed by incubation with a secondary Alexa Fluor Plus 488 labeled goat-anti-mouse antibody (A32723, Invitrogen) and Alexa Fluor Plus 594 labeled goat-anti-rabbit antibody (A32740, Invitrogen) diluted 1:200 (v/v) in PBS-BSAc for 1 h at room temperature. Cells were counterstained with DAPI (0.5 μg/ml; Sigma) for 10 min and mounted using Fluoromount-G (SouthernBiotech, Uden, The Netherlands). A Leica SP5 confocal microscope equipped with a 40 × 1.25 NA Plan-Apochromat oil objective (Leica Microsystems BV, Amsterdam, The Netherlands) was used for imaging. Images were acquired using LAS-AF software (Leica) and processed with Fiji (NIH, Bethesda, MD, USA). The percentage of colocalized AMH and Calreticulin was quantified using Fiji, based on analyzing the Pearson correlation coefficient, as described previously (Adler and Parmryd, 2010). Control cells were incubated with isotype IgG (Cell signaling, Bioke, The Netherlands) instead of the respective primary antibodies, according to the manufacturer’s instructions. Background staining in these controls was negligible.

### Subjects

Thirty-eight age- and BMI-matched subjects of European descent, previously reported in the study of Gorsic *et al.* (2017), were included in this study. Twenty-three subjects were women with PCOS of whom 12 were carriers of an *AMH* variant and 11 were noncarriers. Out of these 12 carriers, 6 were carriers of variants that were selected for functional analysis in this study (three V^12^G carriers, one P^151^S carrier, one P^352^S carrier, one H^506^Q carrier). The other six were carriers of variants which we did not analyze (one A^24^T carrier, one P^46^A carrier, one T^99^S carrier, one R^302^Q carrier, one P^366^L carrier, and one Splicing variant (ex2/3) carrier). Fifteen age- and BMI-comparable control women with regular menses and normal circulating androgen levels were included. PCOS was diagnosed according to the National Institutes of Health (NIH) criteria (elevated levels of total testosterone (T) or non-sex hormone-binding globulin (SHBG)-bound T levels, and chronic oligomenorrhea (eight or fewer menses per year)) (Zawadzki and Dunaif, 1992). Total T, dehydroepiandrosterone sulfate, SHBG, LH, and FSH concentrations were measured in all subjects, as previously reported (Gorsic *et al.*, 2017).

### AMH ELISA assays

Recombinant hAMH in the supernatant and cell lysate samples, and serum AMH levels in 23 PCOS cases and 15 healthy controls, was measured by the picoAMH ELISA assay (Ansh Labs, Webster, TX, USA) and by an automated AMH assay (Lumipulse G1200, Fujirebio Europe). The inter-assay variation was <10% for both assays. The recombinant hAMH measurements reflect the values of pooled samples (n = 4–5) obtained from the stably transfected cells.

### Statistical analysis

For *in vitro* experiments, normality was tested using a Shapiro–Wilk test. Statistical differences were determined by an unpaired parametric *t*-test or by one-way ANOVA parametric test with Dunnett multicomparison using the Prism 9 Software (GraphPad Software Inc., La Jolla, CA, USA). For serum AMH levels, normality was tested using a Shapiro–Wilk test. Serum AMH levels were log-transformed, and next analyzed by AN(C)OVA with adjustment using SPSS Statistics 28 (IBM SPSS, The Netherlands). A *P *<* *0.05 was considered statistically significant. Data are expressed as mean ± SEM.

## Results

### Effect of AMH variants on bioactivity

**Figure 1.**
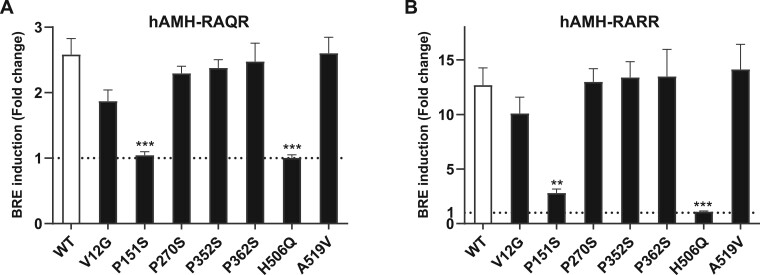
**Analysis of bioactivity of AMH variants.** The mouse granulosa cell line KK-1, stably expressing the anti-Müllerian hormone (AMH) type 2 receptor, were transiently transfected with the AMH-responsive BRE-Luc reporter plasmid together with the AMH variant expression plasmid containing the wild-type cleavage site RAQR (**A**) or the optimized cleavage site RARR (**B**). Luciferase was measured after 24 h incubation. Data are presented as fold change in relative luciferase units expressed relative to the empty vector control (set at 1, represented by the dotted horizontal line). Data points are the mean ± SEM (n = 3 independent experiments performed in triplicate). Statistical differences were analyzed by one-way ANOVA parametric test with Dunnett multicomparison using the Prism 9 Software; **P < 0.01; ***P < 0.001.

Expression of hAMH-^151^S or hAMH-^506^Q, containing the wild-type RAQR cleavage site, failed to induce BRE-Luc reporter activity (Fig. 1A). The presence of the optimized RARR cleavage site did not improve hAMH-^506^Q-induced BRE-Luc activity but resulted in a slight activation by hAMH-^151^S (15% relative to wt-hAMH) (Fig. 1B). Bioactivity of the other five variants was, however, comparable to wt-hAMH. These results were confirmed in the human GC line COV434 cells, although in these cells, wt-hAMH-included BRE-Luc activity was much lower (data not shown).

### Effect of hAMH-^151^S and hAMH-^506^Q on wt-hAMH signaling

Since these *AMH* variants were present in a heterozygous state in patients with PCOS, we further investigated whether hAMH-^151^S and hAMH-^506^Q impacted wt-hAMH bioactivity.

**Figure 2.**
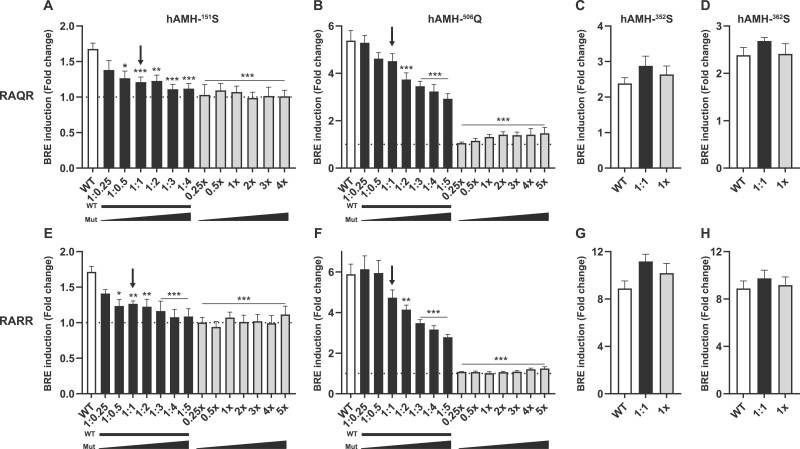
**Inhibitory effect of AMH variants on wt-hAMH bioactivity.** The mouse granulosa cell line KK-1, stably expressing the anti-Müllerian hormone (AMH) type 2 receptor, were transiently transfected with the AMH-responsive BRE-Luc reporter plasmid together with a constant amount of the wt-hAMH expression plasmid and increasing amounts of plasmids encoding P151S (**A**, **E**), H506Q (**B**, **F**), P352S (**C**, **G**), or P362S (**D**, **H**) containing either the wild-type cleavage site RAQR or the optimized cleavage site RARR. Luciferase was measured after 24 h incubation. Data are presented as fold change in relative luciferase units expressed relative to the empty vector control (set at 1, represented by the dotted horizontal line). Data points are the mean ± SEM (n = 3–6 independent experiments performed in triplicate). White bar, wt-hAMH; black bar, wt-hAMH plus hAMH variant; grey bar, hAMH variant. The arrow indicates the 1:1 dosage ratio between wt-hAMH and hAMH variant, reflecting the heterozygous state of the AMH variants. Statistical differences were analyzed by one-way ANOVA parametric test with Dunnett multicomparison using the Prism 9 Software; *P < 0.05; **P < 0.01; ***P < 0.001.

Cotransfection of KK-1 cells with a fixed concentration of the wt-hAMH expression plasmid and increasing amounts of the plasmids expressing hAMH-^151^S or hAMH-^506^Q, resulted in a dose-dependent inhibition of wt-hAMH bioactivity (Fig. 2A–D). At equal concentrations of wt-hAMH and variant AMH expression plasmid transfected, to model the heterozygous state, a 26–28% inhibition of wt-hAMH was observed. This effect was independent of the presence of the RAQR or RARR cleavage site, although in the presence of the RARR cleavage site, higher concentrations of the variant expression plasmids were needed to obtain this inhibitory effect (Fig. 2E and F). In contrast, cotransfection of hAMH-^352^S or hAMH-^362^S with wt-hAMH at an 1:1 gene dosage ratio did not affect wt-hAMH signaling (Fig. 2C, D, G, and H).

### Effect of hAMH-^151^S and hAMH-^506^Q on exogenous wt-hAMH signaling

**Figure 3.**
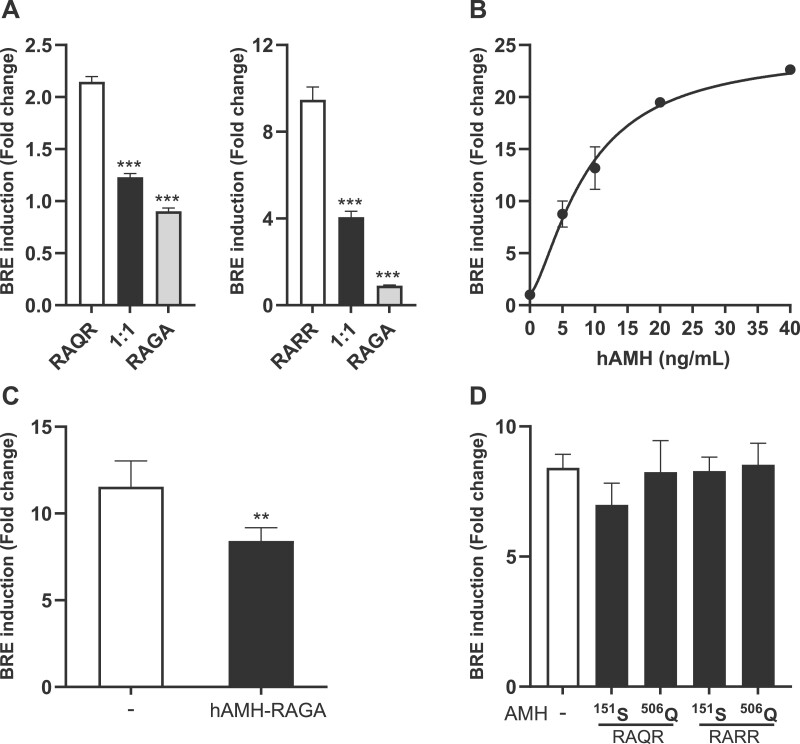
**The effect of hAMH-^151^S and hAMH-^506^Q on exogenous AMH signaling.** (**A**) The mouse granulosa cell line KK-1, stably expressing the anti-Müllerian hormone (AMH) type 2 receptor (KK1/AMHR2), were transiently transfected with the AMH-responsive BRE-Luc reporter plasmid together with an equal amount of plasmids expressing wt-hAMH containing either the wild-type cleavage site RAQR or the optimized cleavage site RARR and wt-hAMH containing the inactive cleavage site RAGA. (**B**) KK1/AMHR2 cells transiently transfected with the AMH-responsive BRE-Luc reporter plasmid were stimulated with increasing concentrations of exogenous recombinant hAMH. (**C**) KK1/AMHR2 cells, transiently transfected with the AMH-responsive BRE-Luc reporter plasmid in absence or presence of an wt-hAMH expression plasmid containing the inactive cleavage site RAGA, were stimulated with 5 ng/ml recombinant hAMH. (**D**) KK1/AMHR2 cells, transiently transfected with the AMH-responsive BRE-Luc reporter plasmid in the absence or presence hAMH-^151^S and hAMH-^506^Q expression plasmids containing either the wild-type cleavage site RAQR or the optimized cleavage site RARR, were stimulated with 5 ng/ml recombinant hAMH. Data are presented as fold change in relative luciferase units expressed relative to the empty vector control. Data points are the mean ± SEM (n = 3–4 independent experiments performed in triplicate). Statistical differences were determined by an unpaired parametric *t*-test (C) or by one-way ANOVA parametric test with Dunnett multicomparison (A and D) using the Prism 9 Software; **P < 0.01; ***P < 0.001.

To further investigate the mechanism of the inhibitory effect of these two variants on wt-hAMH bioactivity, we repeated the experiment using the hAMH-RAGA expression plasmid, which results in a noncleaved AMH protein. Also, when co-transfected with hAMH-RAGA at equal concentrations, wt-hAMH (both RAQR and RARR) bioactivity was significantly decreased, indicating the importance of AMH cleavage (Fig. 3A). To distinguish between a failure in cleavage or secretion, we next investigated whether the AMH variants also affected AMH signaling when wt-hAMH was added exogenously. Exogenous hAMH dose-dependently increased BRE-Luc activity (Fig. 3B). However, BRE-Luc activity induced by 5 ng/ml hAMH was suppressed by 30% (*P* < 0.01) when cells were cotransfected with the hAMH-RAGA plasmid (Fig. 3C). In contrast, exogenous hAMH-induced BRE-Luc activity was not affected when the variants hAMH-^151^S or hAMH-^506^Q were co-transfected (Fig. 3D). These results suggest that AMH-^151^S or AMH-^506^Q display impaired secretion.

### Effect of AMH variants on intracellular biosynthesis and secretion

**Figure 4.**
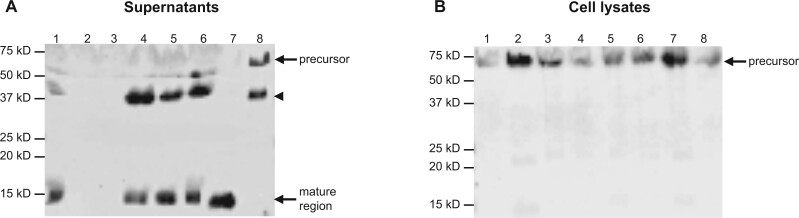
**Western blot analysis of AMH variants.** Western blot analysis of human embryonic kidney epithelial HEK293 cells stably expressing the anti-Müllerian hormone (AMH) variants with the wild-type cleavage site RAQR or the inactive cleavage site RAGA. The mature region-specific 5/6A antibody recognizes the AMH precursor protein (∼75 kD), the cleaved C-terminal mature protein (∼15 kD) and a second subunit owing to a possible second cleavage site (∼40 kDa, indicated by arrowhead). The relative molecular masses (kD) of the protein marker are indicated. Supernatants (**A**) Lane 1: wt-hAMH-RAQR; Lane 2: hAMH-^151^S; Lane 3: hAMH-^506^Q; Lane 4: hAMH-^352^S; Lane 5: hAMH-^362^S; Lane 6: hAMH-^519^V; Lane 7: wt-hAMH-RARR; Lane 8: wt-hAMH-RAGA. Cell lysates. (**B**) Lane 1: wt-hAMH-RAQR; Lane 2: hAMH-^151^S; Lane 3: hAMH-^506^Q; Lane 4: hAMH-^352^S; Lane 5: hAMH-^362^S; Lane 6: hAMH-^519^V; Lane 7: wt-hAMH-RARR; Lane 8: wt-hAMH-RAGA. Supplementary Fig. S1 shows the uncropped western blots.

To analyze the synthesis and secretion of the mutant proteins, western blot analysis was performed using both supernatants and cell lysates of HEK293 cells stably transfected with the AMH variants. In line with the bioactivity experiments, hAMH-^151^S and hAMH-^506^Q proteins could not be detected in the supernatants (Lanes 2 and 3, Fig. 4A), while wt-hAMH, hAMH-^352^S, hAMH-^362^S, and hAMH-^519^V were detected as the cleaved C-terminal mature region of 15 kD, together with an additional band from the potential second cleavage site in the N-terminal pro-region. Also, the noncleaved hAMH-RAGA was detected as a full-length protein in the supernatant (Lane 8, Fig. 4A, Supplementary Fig. S1). However, in the cell lysates, hAMH-^151^S and hAMH-^506^Q were detected as the precursor protein of 75 kD, similar to wt-hAMH (Fig. 4B). These results further indicated a defect in secretion of these two variants.

**Figure 5.**
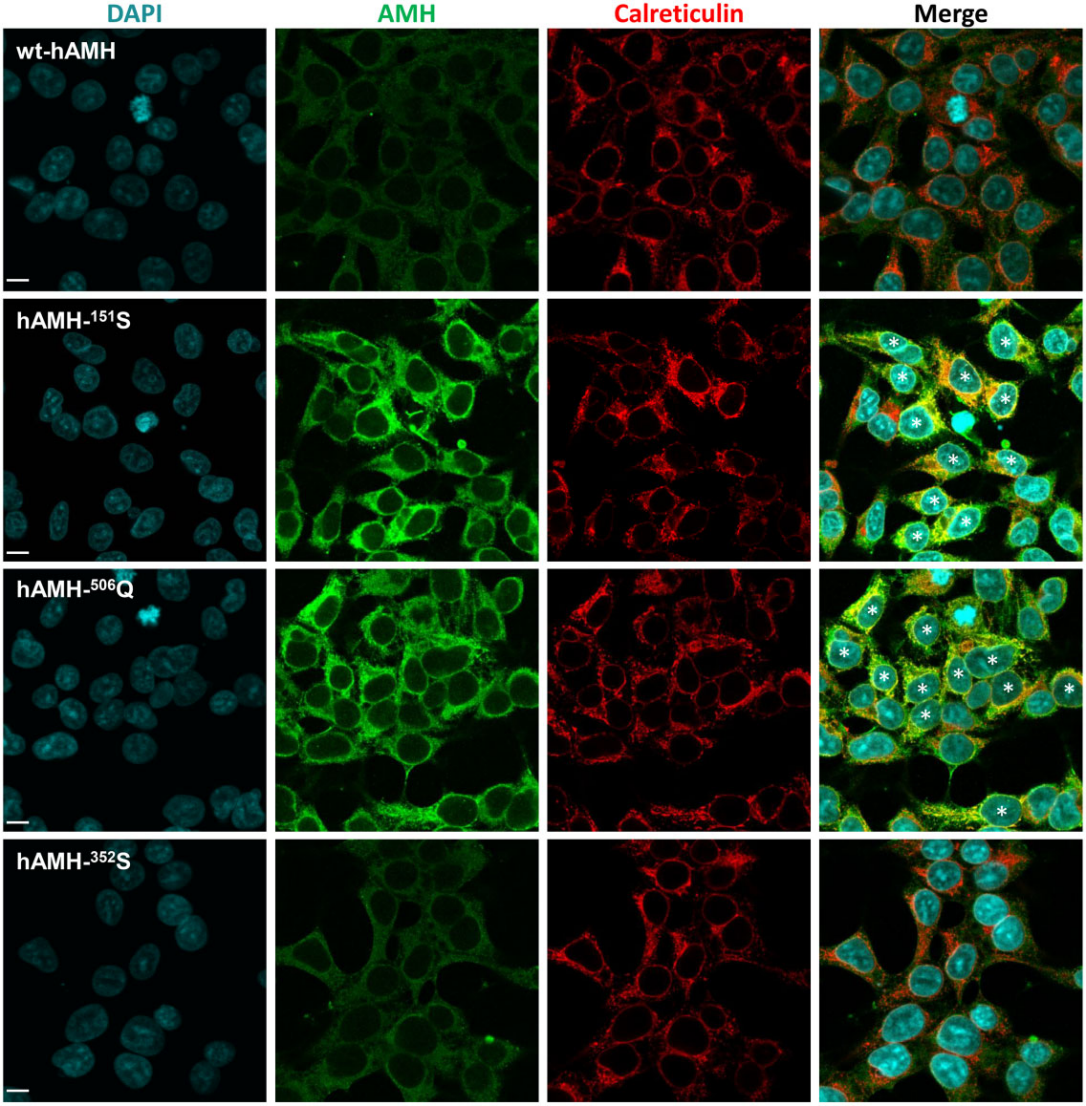
**Cellular localization of AMH variants.** Confocal microscopy was used to detect wt-hAMH, hAMH-^352^S, hAMH-^151^S, and hAMH-^506^Q in stably transfected the human embryonic kidney epithelial HEK293 cells by immunostaining with an anti-Müllerian hormone (AMH) antibody. Double-labeling with reticiculin antibody, as a marker for endoplasmic reticulum, revealed a colocalization in the merge image for hAMH-^151^S and hAMH-^506^Q, but not for wt-hAMH and hAMH-^352^S. The scale bar represents 10 µm. Asterisks indicate co-localization of AMH and calreticulin.

We then used confocal microscopy to visualize the subcellular localization of hAMH-^151^S and hAMH-^506^Q in stably transfected HEK293 cells (Fig. 5). Moderate intracellular AMH staining was observed in cells expressing wt-hAMH and hAMH-^352^S. In contrast, cells expressing hAMH-^151^S and hAMH-^506^Q had a strong intracellular AMH staining.

Double immunofluorescence staining with the endoplasmic reticulum (ER) marker calreticulin revealed a significantly increased colocalization of hAMH-^151^S and hAMH-^506^Q proteins within the ER compared with wt-hAMH (Fig. 5, Supplementary Fig. S2). There was no colocalization with the Golgi (data not shown). These results suggested that these two mutant proteins display an increased ER retention.

### Effect of AMH variants on AMH production and immunoactivity detected by ELISA

To confirm the impaired secretion, we next measured the AMH content in the supernatants and cell lysate of the cells stably transfected with the AMH expression plasmids using the picoAMH ELISA. Consistent with western blot analysis and immunostaining, hAMH-^151^S was detected with high concentrations in the cell lysate but very low levels in the supernatant (Table I). Surprisingly, hAMH-^506^Q and hAMH-^362^S were undetectable in both the cell lysate and supernatant. Moreover, also the hAMH-^519^V variant, identified in control women, was undetectable in the cell lysate and only very low levels were detected in the supernatant. In contrast, both wt-hAMH and hAMH-^352^S were detected at nearly comparable high levels in the supernatant and much lower levels in the cell lysate (Table I). Similar results were obtained for wt-hAMH, hAMH-^151^S, and hAMH-^506^Q with the RARR cleavage site.

**Table I:** Effect of AMH rare variants on AMH production in HEK293 cells.

Cleavage site	Variants	Supernatants* (ng/ml)	Cell lysates* (ng/ml)	Supernatants^#^ (ng/ml)	Cell lysates^#^ (ng/ml)
RAQR	wt-hAMH	10 449.38	15.10	8400	27.3
	hAMH-^151^S	250	153.19	322.3	109.87
	hAMH-^352^S	15 664.14	71.44	9931	67.47
	hAMH-^362^S	Undetectable	Undetectable	9109	51.52
	hAMH-^506^Q	Undetectable	Undetectable	Undetectable	Undetectable
	hAMH-^519^V	33.68	Undetectable	9186	59.31
RARR	wt-hAMH	70 714.87	178.34	25 675	197.56
	hAMH-^151^S	17.03	322.08	92.1	299.3
	hAMH-^506^Q	Undetectable	Undetectable	Undetectable	Undetectable
RAGA	hAMH-RAGA	69 072.83	440.06	35 935	305.5

Anti-Müllerian hormone (AMH) was measured in cell lysates and supernatants of human embryonic kidney epithelial HEK293 cells stably transfected with AMH variants expression constructs with wild-type RAQR cleavage site, the RARR optimized cleavage site, or the inactive cleavage site RAGA. Using the picoAMH assay (Ansh Labs), indicated by *, or the automated Lumipulse G1200 (Fujirebio), indicated by #. Values reflect pooled samples (n = 4–5) obtain from stably transfected cells. RAGA: a cleavage-resistant site.

Since western blot analysis and the picoAMH ELISA yielded discrepant results for hAMH-^362^S, hAMH-^506^Q, and hAMH-^519^V, we questioned whether these variants resulted in a loss of immunoactivity, as we previously reported for AMH-^515^V (Hoyos *et al.*, 2020). Therefore, we re-measured the same samples using the automated Lumipulse G1200, which employs different antibodies. Using this assay, both the hAMH-^362^S and hAMH-^519^V variant were detected at nearly comparable levels as wt-hAMH in the supernatant and cell lysate. Surprisingly, hAMH-^506^Q remained undetectable. The results for hAMH-^151^S were consistent between the picoAMH ELISA and Lumipulse G1200 AMH assay (Table I).

### Circulating AMH levels in women with PCOS harboring AMH variants

AMH levels in sera from women with PCOS who were carriers or noncarriers of AMH protein-altering variants, and control women, were measured using both picoAMH ELISA and automated Lumipulse G1200 (Table II). In line with our previous study (Moolhuijsen *et al.*, 2022), higher AMH levels were measured by the picoAMH ELISA compared to the Lumipulse ELISA, particularly for AMH values in the higher range (results not shown). Carriers of AMH variants had ∼50% lower AMH levels compared to noncarriers, albeit not significantly lower (Table II). Heterozygous carriers of the P^151^S and H^506^Q variants had detectable AMH in both assays. Individual analysis of the AMH variants did not show a clear genotype–phenotype pattern (Table II and Supplementary Table SI).

**Table II:** Circulating AMH levels in controls and women with PCOS with or without heterozygous AMH variants.

	Controls (n = 15)	Non-variant carriers (n = 11)	Variant carriers (n = 12)	*P*-value^a^	*P*-value^b^	*P*-value^c^
Age, years	30.36 ± 1.61	27.67 ± 1.77	27.58 ± 1.75	0.27	0.254	0.97
BMI kg/m^2^	37.62 ± 2.35	36.48 ± 2.54	36.10 ± 2.38	0.747	0.656	0.91
AMH, ng/ml*	3.40 ± 0.69	20.19 ± 6.76	10.07 ± 1.98	<0.001	0.002	0.379
AMH, ng/ml^**#**^	3.26 ± 0.66	16.95 ± 5.66	7.90 ± 1.49	<0.001	0.009	0.303

**AMH levels in individual carriers of studied AMH variants**						
**Variants**	**V12G**	**V12G**	**V12G**	**P151S**	**P352S**	**H506Q**

AMH, ng/ml*	16.97	12.08	6.23	3.04	10.98	5.44
AMH, ng/ml^**#**^	12.06	9.08	5.07	2.72	7.97	4.23

Measurement of serum anti-Müllerian hormone (AMH) by *picoAMH assay (Ansh Labs) or ^#^Lumipulse G1200 (Fujirebio). Data are expressed as mean **±** SEM. *P*-values: ^a^controls versus non-variant carriers; ^b^controls versus variant carriers; ^c^non-variant carriers versus variant carriers.

## Discussion

In this study, we performed extensive functional analyses of *AMH* variants, previously identified in women with PCOS and reproductively normal, control women. *In vitro* analyses showed that hAMH-^151^S and hAMH-^506^Q lack bioactivity and have an inhibitory effect on wt-hAMH signaling. The loss of bioactivity of hAMH-^151^S and hAMH-^506^Q is in agreement with the study of Gorsic *et al.* (2017). However, in contrast to this previous study, the other four tested variants (hAMH-^12^G, hAMH-^270^S, hAMH-^352^S, hAMH-^362^S) all displayed a normal bioactivity. Differences in the cell models used (mouse GC line KK1 and human GC line COV434 versus COS7 cells) may possibly account for differences in AMH processing, as slight differences in bioactivity were observed for hAMH-^151^S when using an optimized cleavage site.

Since these variants were identified as heterozygous variants, it was hypothesized that the mutant protein may exert a dominant negative effect on wild-type AMH (Gorsic *et al.*, 2017). Particularly, mutations that impair the cleavage of TGFβ family members can yield secreted precursors that act as dominant negative mutants (di Clemente *et al.*, 2010). The bio-inactive hAMH-^151^S and hAMH-^506^Q indeed suppressed wt-hAMH signaling in a dose-dependent manner, but we did not observe a clear dominant negative effect at conditions mimicking similar gene dosage. Furthermore, suppression was only observed upon cotranfection of mutant and wt-hAMH but not when cells were stimulated exogenously with wt-hAMH; retention in the ER of the hAMH-^151^S and hAMH-^506^Q proteins causing a failure in secretion explains this differential suppressive effect. The *AMH-H^506^Q* variant has previously also been identified in a male with Persistant Müllerian duct Syndrome (PMDS) (Belville *et al.*, 2004), and similarly, it was shown that AMH-^506^Q was detected in the cell lysate but not in the supernatant of transfected cells (Belville *et al.*, 2004). When proteins do not achieve their native and functional conformation, they are retained in the ER and either undergo retrotranslocation to the cytoplasm for proteasomal or lysosomal degradation or form undegradable protein aggregates (Araki and Nagata, 2012). The colocalization of hAMH-^151^S and hAMH-^506^Q proteins with the ER strongly suggests that these two variant proteins are misfolded and trapped in the ER to form protein aggregates. Thus, the inhibitory effect of these two mutants on wt-AMH signaling likely results from hindering the normal processing and/or secretion of wt-AMH.

Using confocal microscopy, moderate intracellular AMH staining was detected in HEK293 cells expressing wt-hAMH-RAQR, indicative of proper AMH secretion. It has been shown that with the RAQR cleavage site, the cleavage rate of precursor AMH protein can be suboptimal (Nachtigal and Ingraham, 1996). However, the secretion of AMH does not seem to be affected by its proteolytic processing. Here we show that in HEK293 cells stably transfected with the wt-hAMH-RAQR expression plasmid, AMH was detected at high concentrations in the supernatant but relatively low levels in the cell lysate, indicating that the majority of AMH is indeed secreted. Our western blotting results confirm that cleaved AMH isoforms are present in the supernatant, while proAMH was predominantly detected in the cell lysate. We also observed a high concentration of AMH in the supernatant of cells stably transfected with a wt-hAMH plasmid containing the mutated cleavage site RAGA that yields a noncleaved AMH protein. In line with our findings, Belville *et al.* (2004) have previously reported that the secretion rate of an uncleavable AMH variant R^451^T, identified in patients with PMDS, was comparable to wt-AMH protein. These *in vitro* findings are in line with *in vivo* studies, showing that proAMH and the stable noncovalent complex (AMH_N,C_) are both detected in ovarian follicular fluid and serum (Pankhurst *et al.*, 2016; Peigné *et al.*, 2020). Combined, these results suggest that cleavage of AMH takes place upon or after secretion and that AMH secretion is not affected by aberrant cleavage.

The mechanism by which these loss-of-function AMH variants contribute to the PCOS phenotype is not fully clear yet. In analogy to the inhibitory effect of AMH on *CYP17a1* transcription in testicular Leydig cells, Gorsic *et al.* (2019, 2017) hypothesized that reduced AMH signaling might increase *CYP17a1* transcription in theca cells, leading to increased theca cell testosterone production. In support, knockdown of AMH bioactivity in sheep via active immunization resulted in significantly increased androstendione concentrations in ovarian follicular fluid (Campbell *et al.*, 2012) but also increased ovulation rate. On the contrary, based on its role in normal GC, reduced AMH bioactivity may also lead to increased FSH-induced *CYP19* expression, thereby increasing the conversion of androgens into estrogens in GC (Chang *et al.*, 2013). It should be noted that in addition to AMH, other factors also contribute to the inhibition of *CYP19* expression, and thus may overrule the loss of inhibition in the presence of inactive AMH. For example, in cultured human luteinized GCs from normal ovaries, increasing doses of testosterone at concentrations observed in PCOS follicles significantly inhibited both CYP19 mRNA and protein, an effect that was rescued by the androgen receptor antagonist flutamide (Yang *et al.*, 2015). Furthermore, extensive studies have demonstrated impairments in cell viability and growth owing to misfolded proteins (de Vrij *et al.*, 2019). This led us to hypothesize that the abnormal ER retention of the AMH variants P^151^S and H^506^Q might reduce the viability and/or cell proliferation, thereby contributing to the follicular arrest and anovulatory phenotype in PCOS. A potential underlying mechanism may be induction of ER stress. Accumulation of misfolded proteins in the ER causes ER stress and triggers activation of the unfolded protein response (UPR), which in turn affects many cellular functions including apoptosis (Harada *et al.*, 2021). Interestingly, increased expression of *UPR* genes has been observed in GC from antral follicles of women with PCOS (Takahashi *et al.*, 2017). Hence, ER stress has been proposed as a mechanism contributing to the pathology of PCOS (Harada *et al.*, 2021). Whether the AMH variants P^151^S and H^506^Q induce ER stress remains to be determined.

The other four *AMH* variants did not affect intracellular protein synthesis, secretion, and bioactivity. However, we did observe that some of these variants affected the immunoactivity in commercial AMH ELISA kits. The hAMH-^362^S variant, identified in women with PCOS, and the hAMH-^519^V variant, identified in controls without PCOS, both showed severely reduced immunoactivity when measured by the picoAMH ELISA but not by the Lumipulse G1200 AMH ELISA. We recently also reported discordance between immunoactivity and bioactivity for the hAMH-A^515^V variant (rs10417628), which was identified as a homozygous variant in a woman with PCOS (Hoyos *et al.*, 2020). These variants most likely disrupt the antibody-epitope recognition of AMH in the picoAMH ELISA assay. The epitope region 358–369, located in the N-terminal region, is mapped by the capture antibody (Ab24) while the detector antibody (Ab32) has the strongest binding to epitope region 491–502, in the mature domain of AMH (Robertson *et al.*, 2014). The hAMH-P^362^S variant is located in the epitope of the capture antibody while the hAMH-A^519^V variant, similar to hAMH-A^515^V, is close to the epitope of the detector antibody of the picoAMH assay. Surprisingly, hAMH-^506^Q was undetectable by both ELISAs. The hAMH-H^506^Q variant is also very close to the detector antibody epitope of the picoAMH assay. However, since this variant was also not detected by the Lumipulse G1200 assay, additional conformational changes may contribute to the loss of immunoactivity. The Lumipulse assay uses the same antibodies as the Gen II assay (Beckman Coulter, Brea, CA, USA) (Lotierzo *et al.*, 2021), but the exact epitopes are unknown and it has been suggested that the antibodies of this assay recognize conformational epitopes in AMH (Robertson *et al.*, 2014). Since hAMH-^506^Q is retained in the ER, it is very likely that this protein is misfolded, thereby masking its epitopes. Furthermore, H^506^Q is located at the wrist helix consisting of residues 506–514 of the AMH mature domain, which forms the dimer interface between two AMH monomers (Hart *et al.*, 2021). Thus, the change of a histidine at position 506 to glutamine could potentially impair the proper formation of the dimer and thereby contribute to a disrupted conformation of the mutant protein.

Measurement of AMH levels in carriers of the *AMH* variants previously showed that carriers tended to have lower AMH levels than noncarriers (Gorsic *et al.*, 2019). Here we show that between carriers there was, however, a large variation in AMH levels, even between carriers of the same variant (V^12^G). Since in these women PCOS was diagnosed according to the NIH criteria, follicle count is not available. Therefore, in this study, it was not possible to determine whether differences in follicle count explain this variation in AMH levels between carriers, and between carriers and noncarriers. Interestingly, in the carriers of the P^151^S and H^506^Q variant, circulating AMH levels were detectable by both AMH ELISAs. These mutant AMH proteins fail to be secreted *in vitro*. However, it should be noted that these variants are present in a heterozygous state in the women with PCOS, suggesting that wt-AMH is still being produced and secreted. Only in a homozygous state, as shown in a male with PMDS, the H^506^Q variant results in undetectable AMH levels (Belville *et al.*, 2004). AMH levels in carriers of the P^151^S and H^506^Q variants were lower compared to the other variants that showed normal secretion *in vitro*. However, in the absence of follicle numbers, it is difficult to determine whether the mutant protein affects secretion of the wt-AMH protein. Unfortunately, serum from the P^362^S and A^519^V carriers was not available to study the loss of immunoactivity in the picoAMH assay *in vivo*.

In conclusion, our *in vitro* results demonstrate that the PCOS-specific *AMH* variants P^151^S and H^506^Q disrupt the normal processing and secretion of AMH, causing ER retention. In addition, we identified additional AMH variants that impair AMH immunoactivity with (H^506^Q) or without (P^362^S and A^519^V) influencing their bioactivities. Therefore, an *AMH* genetic variant may be considered when serum AMH levels are relatively low in women with PCOS.

## Supplementary Material

gaad011_Supplementary_Data

## Data Availability

The data underlying this article will be shared upon reasonable request to the corresponding author.
