# Supplementary material for: Functional analysis of rare anti-Müllerian hormone protein-altering variants identified in women with PCOS
Source: Mol Hum Reprod. 2023 Apr 2;29(5):gaad011. doi: 10.1093/molehr/gaad011 (PMC10148690; doi:10.1093/molehr/gaad011)
Supplement: gaad011_Supplementary_Data [file gaad011_supplementary_data.pdf]

Supplementary Information

Functional analysis of rare anti-Müllerian hormone protein-altering variants identified in women with PCOS

L. Meng, A. McLuskey, A. Dunaif, J.A. Visser

Contents

Supplementary Table SI Circulating AMH levels in individual carriers of six AMH variants that we did not functionally analyze.

Supplementary Figure S1 Uncropped western blots of AMH variants in HEK293 cells.

Supplementary Figure S2 Colocalization of wt-hAMH, hAMH-^{352}S, hAMH-^{151}S and hAMH-^{506}Q in stably transfected human embryonic kidney epithelial HEK293 cells.

Supplementary Table SI Circulating AMH levels in individual carriers of six AMH variants that we did not functionally analyze.

Variants	A24T	P46A	T99S	R302Q	P366L	Splicing (ex2/3)
AMH, ng/mL*	9.43	3.49	12.23	5.99	27.52	7.43
AMH, ng/mL#	7.89	2.94	10.56	4.87	21.38	6.04

Measurement of serum AMH by * picoAMH assay (Ansh Labs) or # Lumipulse G1200 (Fujirebio).

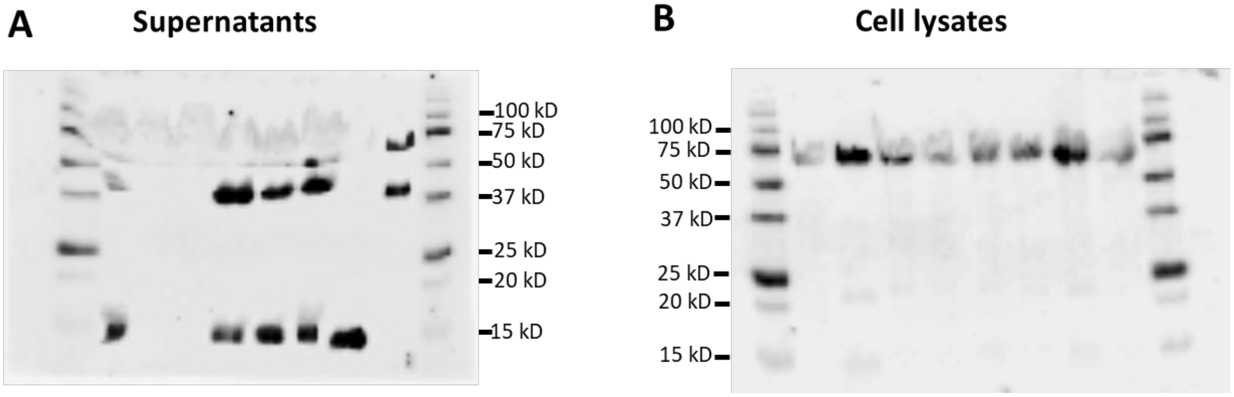

Supplementary Figure S1. **Uncropped western blots of AMH variants in HEK293 cells.**

Western blot analysis of AMH variants: full uncropped blots of Figure 4.

Western blot analysis of the human embryonic kidney epithelial HEK293 cells stably expressing the anti-Müllerian hormone (AMH) variants with the wild-type cleavage site RAQR or the inactive cleavage site RAGA. The mature region-specific 5/6A antibody recognizes the AMH precursor protein (~75 kD), the cleaved C-terminal mature protein (~15 kD) and a second subunit owing to a possible second cleavage site (~40 kDa). The relative molecular masses (kD) of the protein marker are indicated. Supernatants **(A)** Full uncropped blot of Figure 4A. Lane 1: wt-hAMH-RAQR; lane 2: hAMH-^{151}S; lane 3: hAMH-^{506}Q; lane 4: hAMH-^{352}S; lane 5: hAMH-^{362}S; lane 6: hAMH-^{519}V; lane 7: wt-hAMH-RARR; lane 8: wt-hAMH-RAGA. Cell lysates **(B)** Full uncropped blot of Figure 4B. Lane 1: wt-hAMH-RAQR; lane 2: hAMH-^{151}S; lane 3: hAMH-^{506}Q; lane 4: hAMH-^{352}S; lane 5: hAMH-^{362}S; lane 6: hAMH-^{519}V; lane 7: wt-hAMH-RARR; lane 8: wt-hAMH-RAGA.

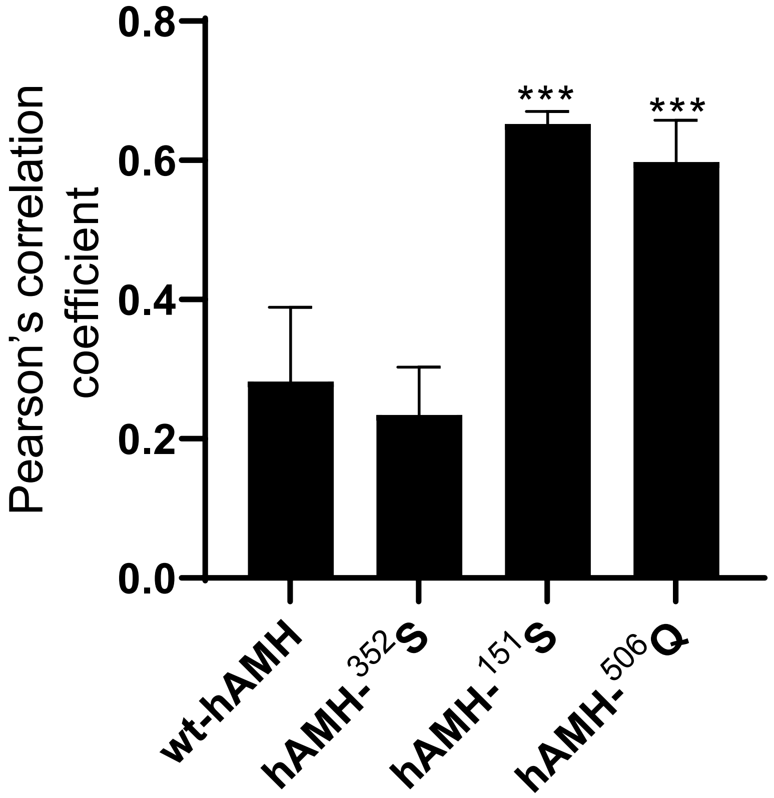

Supplementary Figure S2. Colocalization of wt-hAMH, hAMH-^{352}S, hAMH-^{151}S and hAMH-^{506}Q in stably transfected human embryonic kidney epithelial HEK293 cells.

Colocalization of wt-hAMH, hAMH-^{352}S, hAMH-^{151}S and hAMH-^{506}Q in stably transfected human embryonic kidney epithelial HEK293 cells with endoplasmic reticulum was analyzed by double immunofluorescent staining (confocal microscopy) and measured by Pearson's correlation coefficient (N=6). Statistical differences were analyzed by one-way ANOVA parametric test with Dunnett multi-comparison using the Prism 9 Software; ***P < 0.001
